# The role of Rho kinase (Rock) in re-epithelialization of adult zebrafish skin wounds

**DOI:** 10.1080/21541248.2016.1219208

**Published:** 2016-11-01

**Authors:** Rebecca Richardson, Matthias Hammerschmidt

**Affiliations:** a School of Physiology, Pharmacology & Neuroscience, Faculty of Biomedical Sciences, University of Bristol, Bristol, UK; b Institute of Developmental Biology, University of Cologne, Cologne, Germany; c Center for Molecular Medicine Cologne, University of Cologne, Cologne, Germany; d Cologne Excellence Cluster on Cellular Stress Responses in Aging-Associated Diseases, University of Cologne, Cologne, Germany

**Keywords:** cellular rearrangements, collective cell migration, radial intercalation, Re-epithelialization, Rho kinase, rock, skin, wound healing, zebrafish

## Abstract

Complete re-epithelialization of full-thickness skin wounds in adult mammals takes days to complete and relies on numerous signaling cues and multiple overlapping cellular processes that take place both within the epidermis itself and in other participating tissues. We have previously shown that re-epithelialization of full-thickness skin wounds of adult zebrafish, however, is extremely rapid and largely independent of the other processes of wound healing allowing for the dissection of specific processes that occur in, or have a direct effect on, re-epithelializing keratinocytes. Recently, we have shown that, in addition to lamellipodial crawling at the leading edge, re-epithelialization of zebrafish partial- and full-thickness wounds requires long-range epithelial rearrangements including radial intercalations, flattening and directed elongation and that each of these processes involves Rho kinase (Rock) signaling. Our studies demonstrate how these coordinated signaling events allow for the rapid collective cell migration observed in adult zebrafish wound healing. Here we discuss the particular contribution of Rock to each of these processes.

The process of collective cell migration plays vital roles during several *in vivo* events including wound healing and embryonic morphogenetic movements during development.^1^ These cohesive forms of migration involve the highly coordinated movement of a sheet of epithelial cells and require the individual cells to remain closely connected to maintain epithelial barrier functions and to allow synchronised migration.^1^ Collective cell migration during closure of adult mammalian skin wounds, for example, utilizes similar cellular processes to those required for single cell migration (such as in patrolling immune cells). These include cytoskeletal rearrangements within the leading epidermal cells that allow the formation of anteriorly positioned lamellipodia and filopodia anchoring the cell to an underlying substrate, which, when coupled with retractive cytoskeletal rearrangements at the rear of the cell, facilitate the forward movement of that cell or cells.^2^

The Rho family of GTPases are crucial regulators of actin cytoskeleton dynamics and control lamellipodia and filopodia formation and rear retraction of individual or groups of cells.^2^ Rac and Cdc42 are the predominant family members controlling anterior protrusion assembly whereas RhoA and its downstream mediator Rho-associated kinase (ROCK) are crucial for rear retraction.^2-4^ It has also been demonstrated that Rho and ROCK play a role in the planar cell polarity (PCP) pathway, which regulates epithelial polarity within a sheet of cells and coordinates collective cell migration in multiple developmental contexts.^5-8^ In zebrafish embryos, for example, overexpression of a dominant negative version of Rock2 results in compromised gastrulation convergence extension movements due to impaired cell elongation and orientation, phenocopying PCP component mutants.^7^ Studies involving multiple mutant analyses have also unravelled an essential role of the PCP pathway for embryonic wound closure in mammals.^8^

Recently, we have studied the roles of Rho/Rock, PCP and other pathways during the epidermal re-epithelialization of partial- and full-thickness wounds in adult zebrafish.^9^ The epidermis of late embryonic and larval zebrafish (24 hours post fertilization (hpf) - 15 d post fertilization (dpf)) consists of an inner basal layer attached to the basement membrane and an outer layer of flattened, specialized periderm cells,^10^ resembling the bilayered epidermis of early embryonic mice (E9.5-E12)^11^ and chicks (E6-E14)^12^ in structure and function (Fig. 1A). As development continues, the epidermis in all species thickens to produce additional, intermediary layers between the basal and periderm layers (mouse, E13-E15; chick, E15-E17; zebrafish, 16 dpf-25 dpf).^10-12^ In mouse and chick, terminal differentiation then commences producing a multi-layered, cornified/keratinised epidermis (Fig. 1C).^11,12^ The epidermis of adult zebrafish, by contrast, remains composed of living cells and is similar in structure to the multi-layered, non-keratinised epidermis of late stage embryonic mice and chicks (Fig. 1B).^10-12^

**Figure 1.**
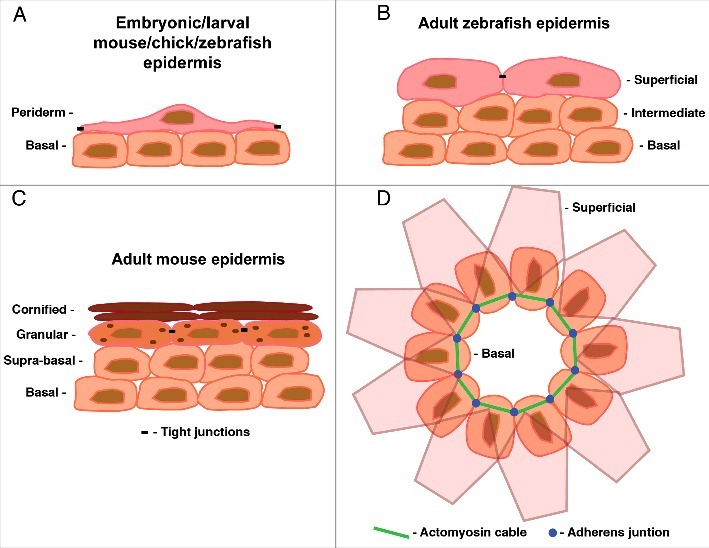
(A-C) Representations of cross sections through the epidermis at different time-points and in the different models used for wound healing studies. (A) In embryonic mice and chicks the epidermis is bilayered at the stages used for embryonic wound closure experiments (E12 in mouse, E6 in chick). At these stages the epidermis consists of an inner basal layer and an outer covering of flattened periderm cells. The epidermis of late embryonic and larval zebrafish is very similar in structure and function. (B) In adult zebrafish the epidermis becomes stratified consisting of 2 to 3 layers of basal and intermediate cells and an outer superficial layer. This resembles the epidermis of mid-gestation mice (E13-E15) and chicks (E15-E17) of corresponding developmental stages. (C) The epidermis of adult mice and chicks is also stratified consisting of 4 distinct layers: the innermost basal layer, the suprabasal layer, the granulation layer where terminal differentiation commences and the outermost cornified layer consisting of terminally differentiated cornified envelopes. (D) Representation of a dorsal view of the bilayered epidermis of an embryonic chick or mouse depicting the actomyosin cable form of wound closure. For simplicity only the LE cells of the basal epidermis are shown. The first row of superficial periderm cells are depicted as semi-translucent to allow vizualization of the basal cells beneath.

Contrary to what is observed in adult mammalian wound healing; re-epithelialization of small wounds in embryonic Drosophila, zebrafish, chick and mammals has been shown to occur via a purse-string mechanism rather than active lamellipodial crawling at the leading edge (LE).^13-19^ This purse-string mechanism involves the rapid formation (within minutes of wounding) of a cable consisting of F-actin and activated myosin-II within the LE basal epidermal cells and allows prompt closure of the wound (Fig. 1D).^13-19^ Rho and ROCK play vital roles during formation of this actomyosin cable in the LE cells, with knockdown of either resulting in a lack of cable formation and consequently a failure of wound healing *in vitro* and *in vivo*.^20-24^ Interestingly, inhibition of ROCK *in vitro* after actomyosin cable assembly has occurred but during cable contraction and wound closure had no affect on re-epithelialization, suggesting that ROCK activity is vital for the formation but not for the function or maintenance of the cable.^23^

Re-epithelialization of cutaneous wounds in adult mammals is a lengthy and step-wise process by comparison, that initiates many hours after wounding and takes 5-7 d to complete.^25-30^ Timely recovery of the wound epidermis is reliant on the proliferative capacity of the keratinocytes, the presence of a suitable substrate for lamellipodial crawling and contraction of the wound edges by the underlying granulation tissue via differentiated myofibroblasts.^25-30^ By contrast, the rapid re-epithelialization we observed in both full-thickness wounds (∼2 mm in diameter and down to the level of the sub-cutaneous adipocytes; ∼250 µm/h) and partial-thickness wounds (∼0.5 mm in diameter and retaining the basal dermal layer; ∼500 µm/h) in adult zebrafish resembles what is observed in embryonic mammals in it's timing and lack of reliance on other wound healing processes.^9,31^ Due to this rapid nature of re-epithelialization in adult zebrafish, with no apparent initial lag phase of forward movement, we sought to determine the presence of purse-string mechanisms in our injury models. Using live imaging of adult transgenic zebrafish and labeling of actin and myosin in fixed samples we could observe the formation of a purse-string in very small wounds (2-3 cell diameters) but not following our full- or partial-thickness injuries.^9^ This suggests that in larger wounds, purse-string mechanisms are, if at all, only involved in the final steps of wound closure, when the area yet to be re-covered by keratinocytes has become very small and the number of LE keratinocytes has been reduced to a few cells.

Due to the established role of Rho and ROCK in collective cell migration we hypothesized that they may also be required during adult zebrafish re-epithelialization. Indeed, chemical inhibition of Rock via Y27632 or the Rho kinase inhibitor III, Rockout, resulted in significantly slower re-epithelialization of full- and partial-thickness wounds in adult zebrafish.^9^ As we have shown that purse-string mechanisms do not play a predominant role in the closure of larger adult zebrafish injuries, this would suggest that other Rock-dependent processes must be affected and are required for closure of these wounds. Via time-lapse imaging of live double transgenic fish following partial-thickness wounding we could reveal that the basal-most keratinocytes at the LE extended lamellipodia while superficial cells lagged behind, suggesting an active migratory mode of re-epithelialization similar to adult mammals (Fig. 2A).^9^ Yet, unlike in adult mammals, this active migration occurs without any apparent lag phase (lamellipodia were observed within minutes of wounding) and at significantly higher speeds than recorded for other models.^9^ Epithelial cells *in vitro* also close scratch wounds via lamellipodial cell crawling and several rows of following epithelial cells are also observed to produce (cryptic) lamellipodia during collective cell migration.^32-34^ We observed a similar phenomenon in second and third row keratinocytes following the LE in zebrafish wounds *in vivo* (Fig. 2A).^9^ However, inhibition of Rock had no effect on these LE or cryptic protrusions apart from slightly randomizing their spatial orientation, most likely due to the role of Rock in the PCP pathway (see above and below) (Fig. 2A).^9^ Thus, LE cells sometimes produced more than one major protrusion that projected more randomly and not necessarily directly toward the wound bed.

**Figure 2.**
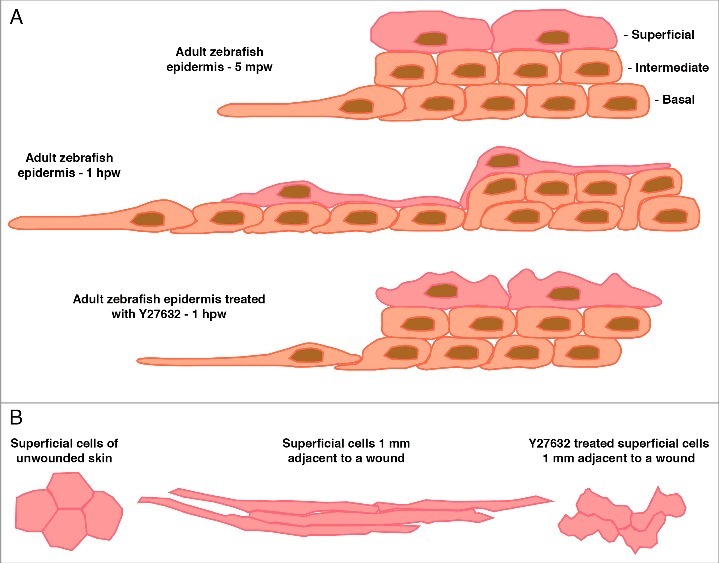
(A) Representation of a cross section through the epidermis at the wound edge in an adult zebrafish 5 minutes post-wounding (mpw), one hour post-wounding (hpw), or 1 hpw following Rock inhibition. The epidermis initially consists of 2-3 layers of inner basal and intermediary cells and a layer of superficial cells. Within 5 mpw, the LE basal cells have begun to make lamellipodial protrusions and elongate onto the denuded area. By 1 hpw directed radial intercalations of intermediary cells into the basal layer have occurred producing a single layer of inner cells; the superficial cells have elongated to ensure coverage of the increased basal layer area; the LE cells and several cells behind are still protrusive and have undergone some cell elongation, collectively allowing the whole epidermal sheet to move forward to cover the wound. Following Rock inhibition the processes of radial intercalation and coordinated elongation are severely affected resulting in reduced forward movement even though LE protrusions are maintained. (B) Dorsal view outlines of 4 individual superficial cells taken from images from adult zebrafish at the indicated distance from a full-thickness wound (wound is to the left). In unwounded epidermis, the superficial cells are hexagonal in shape. Following wounding the superficial cells elongate in the direction of the wound to up to 10-times their original diameter. Following Rock inhibition the superficial cells change shape but in an uncoordinated manner resulting in many random shapes.

A much more striking effect on LE protrusions was obtained upon inhibition of TGFβ signaling or interference with integrin-ECM binding, which compromised lamellipodial shape and stability, as well as re-epithelialization rates.^9^ TGFβ has been shown to induce a more migratory phenotype in mammalian keratinocytes by inducing the expression of integrins α5β1, αvβ5 and αvβ6 within anterior protrusions.^35-37^ However, it has also been suggested that TGFβ has a counteractive negative effect on re-epithelialization by suppressing keratinocyte proliferation, and that this effect is predominant, leading to a net increase in re-epithelialization rates upon genetically compromised TGFβ signaling.^25,37,38^ However, direct *in vivo* evidence for such counteractive TGFβ roles is difficult to obtain due to the overlapping time-scale of keratinocyte proliferation and migration during adult mammalian wound healing.^25,37,38^ By contrast, re-epithelialization of adult zebrafish wounds is independent of keratinocyte proliferation, indicated by its insensitivity to inhibition of DNA replication, allowing independent examination of the effect of TGFβ inhibition on lamellipodial crawling of basal keratinocytes.^9^ Due to the similar results we obtained from inhibiting TGFβ signaling or integrin-ECM interactions, we hypothesized that, consistent with the studies in mammalian wound healing systems, TGFβ signaling promotes LE protrusion stability and functionality by modifying integrin populations at the basal surface of LE keratinocytes, thereby allowing a better and more dynamically regulated adhesion of protrusions with the migration substrate.^9,37^ Consistent with this notion, the effect on protrusion stability and functionality only compromised closure rates of partial-thickness wounds where a dermal substrate is still present. In turn, this suggests that other cellular mechanisms are more important for full-thickness wound re-epithelialization. Furthermore, in light of the absence of purse-string mechanisms during most phases of wound re-epithelialization, and the dispensability of Rock signaling for lamellipodial crawling at the LE (see above), we reasoned that these other cellular mechanisms might be the main Rock targets underlying the ∼4-fold reduction in the closure rate of full-thickness wounds upon Y27632 treatment.^9^

Live imaging of the entire re-epithelialization process following full-thickness wounding revealed cellular events within the intact epidermis at some distance from the wound.^9^ Indeed, close examination of the epidermis up to 2 mm away from the wound revealed that the rapid re-epithelialization observed in adult zebrafish is a result of multiple coordinated epithelial rearrangements in regions behind the LE, including radial intercalations and directed and coordinated cell flattening, polarization and elongation (Fig. 2).^9^ Initially, basal and intermediary keratinocytes behind the LE undergo radial intercalations leading to a long-range progressive reduction of cell layers and a concomitant lateral extension of the remaining basal-most layer (Fig. 2A).^9^ This partly provides the additional keratinocytes required to cover the wound and is a process that is independent of cell proliferation and similar to events immediately adjacent to the LE described in other wound healing models.^39^ Blocking Rock function with Y27632 led to severely compromised keratinocyte flattening and a failure of radial intercalations within the epidermis around the full-thickness wounds, paired with improper localization of non-muscle myosin within the inner keratinocytes (Fig. 2A).^9^ This is likely to limit the capacity for forward movement of the collective cell sheet, underlying the strong reduction in wound closure rates (Fig. 2A). These findings are in line with previous reports that Rock drives cell flattening during epidermal stratification and spreading in mammals and radial cell intercalations during gut morphogenesis in amphibia.^40-42^

The following keratinocytes also undergo directed and coordinated planar polarization and elongation, predominantly in the superficial layer, which apparently does not participate in the underlying intercalation movements, but also to a lower extent in intermediary layers (Fig. 2).^9^ Indeed, superficial cells elongate up to 10-times their original diameter (Fig. 2B).^9^ Similar superficial cell elongations have been reported during wound closure in mouse, zebrafish and Drosophila embryos.^16,43-45^ Following inhibition of Rock we also observed a failure in this directed superficial cell elongation, with cells managing to change their shape but in a random and uncoordinated manner (Fig. 2B).^9^ These findings would support a role for Rock in the PCP pathway to allow coordinated elongations and are similar to previously described effects of Rock perturbation during zebrafish and mouse development.^7,41^ However, transgenic blockage of the PCP pathway did not affect wound closure to the same extent as the inhibition of Rock, although both of these treatments had comparable effects on directed keratinocyte elongation.^9^ This suggests that the PCP pathway coordinates cell elongation but, unlike Rock, is not required for flattening and radial intercalations. In turn, this means that during the latter, and more crucial, epithelial rearrangements, Rock must be part of other pathways that have not been elucidated in this context as yet. Interestingly, a recent report suggests that ROCK functions downstream of the PCP component Celsr1 during mouse epidermal radial intercalations but that this signaling axis may be separate from other PCP driven developmental processes.^41^ Collectively, our data indicate that the coupling of basal/intermediary radial intercalation and cell flattening and elongation, both reliant on Rock function, within the following keratinocytes is vital for, and is likely to be the driving force behind, the rapid re-epithelialization characteristic of adult zebrafish. In contrast, the PCP- and Rock-dependent directionality of cell elongations only has a minor impact on wound re-epithelialization, possibly by indirectly promoting radial cell intercalations that would be compromised if cells elongated in random directions. Of note, cell flattening and radial intercalation are also major driving forces of epiboly, one of the morphogenetic processes during gastrulation,^46^ which also, at least partly, depends on Rho function.^21^ Actually, the term “epiboly” was initially introduced in the context of wound closure,^47^ and was later adopted by developmental biologists in the context of gastrulation without knowing at the time of the similarities in the underlying cellular rearrangements and their genetic control.

Future studies have to address the presence and impact of such Rock-dependent long-range epithelial rearrangements during wound re-epithelialization in mammals. In addition, while our data suggest that Rock regulates these epithelial rearrangements via the actomyosin system, the regulators upstream of Rho/Rock in this context remain largely unclear. Most Rho GTPases cycle between a GTP-bound active conformation and a GDP-bound inactive conformation, mediated by guanine nucleotide exchange factors (GEFs) promoting the active and GTPase-activating proteins (GAPs) promoting their inactive state.^48,49^ In addition, Rho proteins and GEFs are regulated via post-translational modifications and their subcellular distribution, triggered by various cell-surface receptors including integrins, cadherins, cytokine receptors, receptor tyrosine kinases (RTKs) and G protein-coupled receptors.^48,49^ Our analyses have largely ruled out integrins and the RTKs of the FGFR family as such stimulators.^9^ Once identified, the relevant cell surface receptors might serve as targets for pharmacological intervention with corresponding ligand agonists, allowing faster wound closure by accelerating the recruitment of re-epithelializing keratinocytes from skin regions around the wound. As in adult zebrafish, such improvements would occur at the expense of the thickness of the adjacent epidermis, however, this should be a minor problem, as keratinocyte proliferation after the wound has closed could re-establish the epidermis to its normal thickness, as is the case in adult zebrafish.^9^

## Abbreviations

ROCK/Rock: Rho associated kinase
PCP: Planar cell polarity
LE: Leading edge
TGF β: Transforming growth factor β
ECM: Extracellular matrix
hpf: hours post fertilization
dpf: days post fertilization
hpw: hours post wounding
